# Introduction and expansion of the SARS-CoV-2 B.1.1.7 variant and reinfections in Qatar: A nationally representative cohort study

**DOI:** 10.1371/journal.pmed.1003879

**Published:** 2021-12-16

**Authors:** Laith J. Abu-Raddad, Hiam Chemaitelly, Houssein H. Ayoub, Peter Coyle, Joel A. Malek, Ayeda A. Ahmed, Yasmin A. Mohamoud, Shameem Younuskunju, Patrick Tang, Zaina Al Kanaani, Einas Al Kuwari, Adeel A. Butt, Andrew Jeremijenko, Anvar Hassan Kaleeckal, Ali Nizar Latif, Riyazuddin Mohammad Shaik, Hanan F. Abdul Rahim, Gheyath K. Nasrallah, Hadi M. Yassine, Mohamed Ghaith Al Kuwari, Hamad Eid Al Romaihi, Mohamed H. Al-Thani, Abdullatif Al Khal, Roberto Bertollini

**Affiliations:** 1 Infectious Disease Epidemiology Group, Weill Cornell Medicine–Qatar, Cornell University, Doha, Qatar; 2 World Health Organization Collaborating Centre for Disease Epidemiology Analytics on HIV/AIDS, Sexually Transmitted Infections, and Viral Hepatitis, Weill Cornell Medicine–Qatar, Cornell University, Doha, Qatar; 3 Department of Population Health Sciences, Weill Cornell Medicine, Cornell University, New York, New York, United States of America; 4 Mathematics Program, Department of Mathematics, Statistics, and Physics, College of Arts and Sciences, Qatar University, Doha, Qatar; 5 Hamad Medical Corporation, Doha, Qatar; 6 Biomedical Research Center, QU Health, Qatar University, Doha, Qatar; 7 Wellcome–Wolfson Institute for Experimental Medicine, Queens University, Belfast, United Kingdom; 8 Genomics Laboratory, Weill Cornell Medicine–Qatar, Cornell University, Doha, Qatar; 9 Department of Genetic Medicine, Weill Cornell Medicine–Qatar, Cornell University, Doha, Qatar; 10 Department of Pathology, Sidra Medicine, Doha, Qatar; 11 College of Health Sciences, QU Health, Qatar University, Doha, Qatar; 12 Department of Biomedical Science, College of Health Sciences, QU Health, Qatar University, Doha, Qatar; 13 Primary Health Care Corporation, Doha, Qatar; 14 Ministry of Public Health, Doha, Qatar; Universitair Medisch Centrum Utrecht, NETHERLANDS

## Abstract

**Background:**

The epidemiology of the SARS-CoV-2 B.1.1.7 (or Alpha) variant is insufficiently understood. This study’s objective was to describe the introduction and expansion of this variant in Qatar and to estimate the efficacy of natural infection against reinfection with this variant.

**Methods and findings:**

Reinfections with the B.1.1.7 variant and variants of unknown status were investigated in a national cohort of 158,608 individuals with prior PCR-confirmed infections and a national cohort of 42,848 antibody-positive individuals. Infections with B.1.1.7 and variants of unknown status were also investigated in a national comparator cohort of 132,701 antibody-negative individuals. B.1.1.7 was first identified in Qatar on 25 December 2020. Sudden, large B.1.1.7 epidemic expansion was observed starting on 18 January 2021, triggering the onset of epidemic’s second wave, 7 months after the first wave. B.1.1.7 was about 60% more infectious than the original (wild-type) circulating variants. Among persons with a prior PCR-confirmed infection, the efficacy of natural infection against reinfection was estimated to be 97.5% (95% CI: 95.7% to 98.6%) for B.1.1.7 and 92.2% (95% CI: 90.6% to 93.5%) for variants of unknown status. Among antibody-positive persons, the efficacy of natural infection against reinfection was estimated to be 97.0% (95% CI: 92.5% to 98.7%) for B.1.1.7 and 94.2% (95% CI: 91.8% to 96.0%) for variants of unknown status. A main limitation of this study is assessment of reinfections based on documented PCR-confirmed reinfections, but other reinfections could have occurred and gone undocumented.

**Conclusions:**

In this study, we observed that introduction of B.1.1.7 into a naïve population can create a major epidemic wave, but natural immunity in those previously infected was strongly associated with limited incidence of reinfection by B.1.1.7 or other variants.

## Abstract 2

Laith Abu-Raddad and colleagues describe the introduction and expansion of the SARS-CoV-2 B.1.1.7 variant in a national cohort in Qatar.

## Author summary

**Why was this study done?:**

- A novel SARS-CoV-2 variant emerged in the United Kingdom, B.1.1.7 (known also as Alpha), but the consequences of the introduction of this variant into a new national population are insufficiently understood.
- The first study objective was to characterize the epidemiology of B.1.1.7 immediately after its introduction into a naïve population for this variant.
- The second study objective was to investigate reinfections with B.1.1.7 and to estimate the efficacy of natural infection against reinfection with this variant.

**What did the researchers do and find?:**

- We investigated the epidemiology of B.1.1.7 using 2 national retrospective cohort studies, mathematical modeling, and other statistical analyses to answer the study’s research questions.
- A sudden, large, and rapidly growing epidemic wave of B.1.1.7 cases commenced shortly after introduction of this variant into Qatar, and findings suggest that B.1.1.7 was approximately 60% more infectious than the original wild-type circulating variants.
- The efficacy of natural infection against reinfection with B.1.1.7 was estimated at 97.5% (95% CI: 95.7% to 98.6%) among those with a prior PCR-confirmed infection and at 97.0% (95% CI: 92.5% to 98.7%) among those with a prior antibody-positive result.

**What do these findings mean?:**

- Given its infectiousness, the B.1.1.7 variant can spark an epidemic wave once introduced into a naïve population, even in the presence of public health restrictions and high levels of natural immunity.
- Prior infection with a wild-type SARS-CoV-2 variant is associated with strong protection against infection with B.1.1.7, a variant that does not appear to be associated with serious immune evasion.

## Introduction

Qatar had a significant first wave of the severe acute respiratory syndrome coronavirus 2 (SARS-CoV-2) epidemic that peaked in late May 2020 [1,2]. Public health restrictions were imposed following the diagnosis of the first infection in Qatar in early March 2020, and included a range of measures that severely affected mobility and social and economic activities [1–3]. These restrictions included partial lockdowns, school closures and online schooling, mask mandates, and travel restrictions. The restrictions were tightened or eased at different times depending on the status of the epidemic and the need to reduce infection transmission [2,4]. Qatar also launched a mass coronavirus disease 2019 (COVID-19) immunization campaign on 21 December 2020, using the BNT162b2 (Pfizer–BioNTech) [5] and mRNA-1273 (Moderna) [6] vaccines, but vaccine coverage was limited before the end of March 2021 [7–9].

The first wave in Qatar has been thoroughly investigated and featured 2 subepidemics [1,2,4,10–18]. The first subepidemic emerged among craft and manual workers [1,3]. These workers constitute 60% of the population and are mostly single young men who typically live in large shared accommodations [19,20]. In a nationwide population-based sero-survey, nearly 6 out of every 10 workers had detectable antibodies against SARS-CoV-2 [13]. The second subepidemic occurred among the “urban population” that constitutes the remaining 40% of the population [1]. The individuals in this population are typically people employed in the professional or service sectors, who reside in single-unit and family households, including children, adults, and/or older adults [19,21]. Until the introduction of the B.1.1.7 variant, this subepidemic remained at low incidence, with fewer than 2 of every 10 individuals having detectable antibodies against SARS-CoV-2 [18].

Qatar has advanced digital healthcare and surveillance systems. Given the global concern about novel SARS-CoV-2 variants [22], a surveillance system was set up to track the entry and expansion of these variants in Qatar. The objectives of this study were to (i) investigate the epidemiology of B.1.1.7 (the Alpha SARS-CoV-2 variant) [23], first detected in the United Kingdom [22], as this variant entered and expanded in the population, and (ii) assess reinfections with B.1.1.7 in national cohorts of those with a prior PCR-confirmed infection and those already antibody-positive, relative to infections in a comparator cohort of antibody-negative individuals.

## Methods

### Sources of data

This study was conducted in the resident population of Qatar. The study did not have a specific prior prospective protocol, but was conducted as informed by the methods and analysis plans of our previous work [1,16,17].

We analyzed the centralized national SARS-CoV-2 databases compiled at Hamad Medical Corporation (HMC), the main public healthcare provider and the nationally designated provider for all COVID-19 healthcare needs. These databases have captured SARS-CoV-2-related data since the start of the epidemic, such as all records of polymerase chain reaction (PCR) testing, COVID-19 hospitalizations, infection severity classification per World Health Organization (WHO) guidelines [24] (performed by trained medical personnel through individual chart reviews), and COVID-19 deaths, also assessed per WHO guidelines [25]. These databases are complete, with no missing information for PCR testing, COVID-19 hospitalizations, and basic demographic details since the start of the epidemic. The databases also cover most serological testing for SARS-CoV-2 conducted in Qatar, including both testing done on residual blood specimens collected from attendees at HMC for routine clinical care [18] and testing during a series of population-based serological surveys [13,14].

All PCR tests conducted in Qatar are classified on the basis of symptoms and reason for testing (clinical suspicion [presence of symptoms compatible with a respiratory tract infection], contact tracing, random testing campaigns [or surveys with no further specification], individual request, routine healthcare testing, pre-travel, at port of entry, or other). Qatar has young and diverse demographics. Only 9% of its residents are ≥50 years of age, and 89% are expatriates, most of whom are male, working in Qatar from over 150 countries [1,20].

### Laboratory methods and viral genome sequencing and analysis

SARS-CoV-2 PCR and antibody testing methods are described in Text A in S1 File. All nasopharyngeal and/or oropharyngeal swabs were collected by healthcare professionals. All tests were processed at the HMC Central Laboratory or Sidra Medicine Laboratory, following standardized protocols. Viral genome sequencing was conducted on a subset of S-gene “dropout” samples to confirm B.1.1.7. The methods of the viral genome sequencing are described in Text B in S1 File.

### Surveillance system for B.1.1.7

A surveillance system was set up to track the entry and expansion of variants of concern in Qatar and to confirm variant community transmission via real-time quantitative reverse transcription PCR (RT-qPCR) genotyping and viral genome sequencing [11,26–28]. A component of the RT-qPCR surveillance included identifying B.1.1.7 using the TaqPath COVID-19 Combo Kit platform (Thermo Fisher Scientific [29]). This platform is employed in >85% of all PCR testing conducted in Qatar and co-amplifies the viral S, N, and ORF1ab regions. Informed by available evidence [30–32], a B.1.1.7 case was proxied as an S-gene “dropout” (or “target failure”) case, using stringent quantitative criteria of (i) PCR cycle threshold (Ct) value ≤ 25 for both the N and ORF1ab genes and (ii) a negative outcome, or Ct value ≥ 32, for the S gene, to prevent false-positive B.1.1.7 cases. The validity of this definition was demonstrated previously [32,33], and also validated in Qatar in the present study through viral genome sequencing (see below). With the PCR Ct value being ≤25 for both the N and ORF1ab genes, this definition ensures that infections and reinfections with this variant are acute recent infections [34–36].

### Effective reproduction number and infectiousness of B.1.1.7

Mathematical modeling was used to estimate the effective reproduction number (*R*_*t*_), that is, the number of secondary infections each infection generated at time *t*. *R*_*t*_ was estimated for both SARS-CoV-2 infection by any variant and SARS-CoV-2 infection by B.1.1.7, using the Robert Koch Institute method [4,37,38].

*R*_*t*_ is expressed in terms of the social contact rate in the population (*c*; how often people meet), the likelihood of the virus to be transmitted (which depends on viral load) in 1 contact between an infectious individual and a susceptible individual (*p*; transmission probability of the infection per contact), duration of infectiousness (*D*; duration over which an infected individual can pass the infection to another individual), and proportion of the population that is still susceptible to the infection (*S*; proportion not yet immune to it): *R*_*t*_ = *c* × *p* × *D* × *S*.

**Fig 1:**
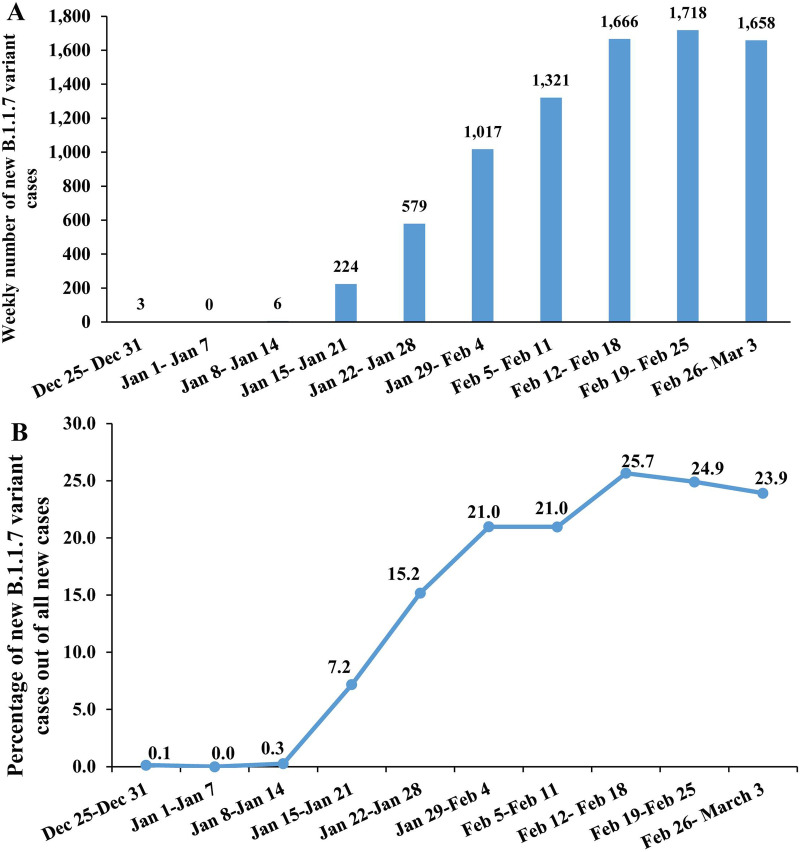
SARS-CoV-2 B.1.1.7 variant expansion in Qatar. (A) Time series of the number of new B.1.1.7 cases. (B) Percentage of new B.1.1.7 variant cases among all PCR-confirmed cases.

Since the terms *c* and *S* are essentially stable over a duration of a few weeks and could not have changed suddenly at the onset of B.1.1.7 expansion (18 January 2021; Fig 1), a sudden change in *R*_*t*_ immediately following B.1.1.7 expansion would reflect a change in the overall infectiousness of the virus, that is, a change in *p* and/or *D*. The overall relative infectiousness of B.1.1.7 can thus be estimated by comparing *R*_*t*_ before the expansion of this variant, to $R_{t}^{B.1.1.7}$, the effective reproduction number of only this variant after its expansion in the population.

**Fig 2:**
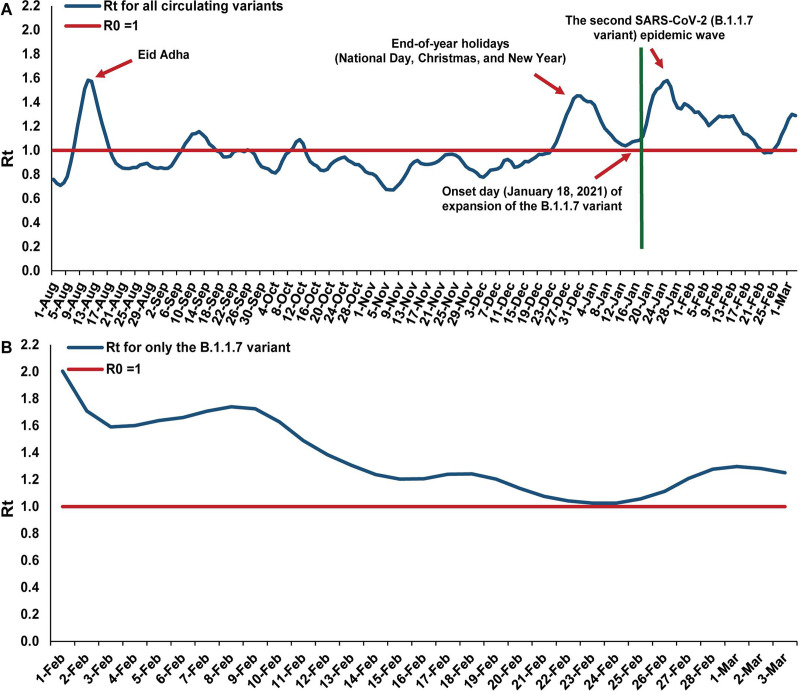
Estimated effective reproduction number (*R*_*t*_) for SARS-CoV-2 infection. (A) All circulating variants. (B) Only B.1.1.7. *R*_t_ was calculated using the Robert Koch Institute method [37].

The relative infectiousness of B.1.1.7 compared to the original circulating variants was estimated by dividing the average $R_{t}^{B.1.1.7}$ in the third and fourth weeks of its expansion (1–14 February 2021) by the average *R*_*t*_ prior to the B.1.1.7 expansion (during the overall stable incidence phase extending from 1 August 2020 to 17 January 2021; Fig 2). During this time, and more broadly from 1 August 2020 to end of this study on 3 March 2021, there was no major change in public health restrictions in Qatar [4], and COVID-19 vaccine coverage was very limited [7–9]. The first 2 weeks of B.1.1.7 expansion were not used in the calculation because the numbers were unstable, as transmission appears to have been influenced by 1 or more superspreading events that were not representative of the average community transmission. Week 5 and weeks thereafter were not used in the calculation to avoid biasing the estimate by depletion of susceptible individuals [39].

### Associations of individual characteristics with infection with B.1.1.7

Associations of individual characteristics with PCR positivity for B.1.1.7 infection were explored using chi-squared tests and univariable logistic regressions. Odds ratios (ORs), 95% confidence intervals (CIs), and *p*-values are reported. The statistical analysis plan was to use variable selection, but in practice no variable selection was needed. All variables had a 2-sided *p*-value ≤ 0.2 in univariable regression analysis and hence were eligible for inclusion in the multivariable analysis to estimate adjusted ORs and associated 95% CIs and *p*-values. Variables with 2-sided *p*-value ≤ 0.05 in the multivariable model were considered associated with infection with B.1.1.7.

### Assessment of reinfection with B.1.1.7 and variants of unknown status

We designed 2 retrospective cohort studies to estimate the efficacy of natural infection against reinfection with B.1.1.7 as a primary outcome. The first study compared the incidence of reinfection in the national cohort of individuals with a prior PCR-confirmed infection before 1 November 2020 to the incidence of infection in the national cohort including all antibody-negative individuals who had no evidence of prior infection, whether ascertained by PCR or antibody testing, before study onset (18 January 2021, the onset date of the B.1.1.7 expansion; Fig 1). The 1 November 2020 cutoff date was chosen to avoid misclassification of prolonged PCR-positive cases as reinfections [34,40–43].

The second cohort study compared the incidence of reinfection in the national cohort of individuals with a documented antibody-positive record before 1 January 2021 to incidence of infection in the national cohort of antibody-negative individuals with no evidence of any prior infection. The 1 January 2021 cutoff date was chosen to exclude cases in which antibody testing and PCR testing were done at about the same time as part of the clinical care of COVID-19 patients—a PCR-positive swab within a few days of an antibody-positive test may reflect an active primary infection under clinical consideration rather than a reinfection [17].

Follow-up in both studies was from 18 January 2021 to 3 March 2021. Individuals in both studies were followed until documented infection (with B.1.1.7 or a variant of unknown status), death, or end-of-study censoring. Outcomes of all PCR tests in Qatar during the study duration were included, regardless of the reason for PCR testing or presence of symptoms.

Frequency distributions and measures of central tendency were used to characterize study cohorts, as well as episodes of reinfections and of primary infections in these cohorts. Standardized mean differences (SMDs) were used to compare groups, with SMD ≥ 0.1 indicating evidence for differences [44].

### Reinfection risk and rate

Cumulative risk was defined as the proportion of individuals identified with a reinfection/infection with a variant of interest during the study period among all eligible individuals in each cohort.

The incidence rate of documented reinfection/infection in each cohort was calculated by dividing the number of reinfection/infection cases identified during the study for a variant of interest by the number of person-weeks contributed by all eligible individuals in the cohort. The incidence rate was estimated using a Poisson log-likelihood regression model with the STATA 16.1 [45] *stptime* command.

### Comparator antibody-negative group and efficacy of natural infection against reinfection

Incidence of infection with B.1.1.7 and variants of unknown status in the national cohort of individuals who were antibody-negative before 1 January 2021 and did not have a PCR-confirmed infection before study onset (18 January 2021) was compared with the incidence of reinfection with B.1.1.7 and variants of unknown status in the PCR-confirmed and antibody-positive cohorts. The risk of documented infection and the incidence rate of documented infection in the antibody-negative cohort were assessed using the same approach used for the other 2 cohorts.

The efficacy of natural infection against reinfection was estimated by comparing the incidence rate of reinfection in the PCR-confirmed cohort (and antibody-positive cohort) to the incidence rate of infection in the comparator antibody-negative cohort:

$$efficacy\;against\;reinfection=1-\frac{incidence\;rate\;of\;reinfection\;in\;the\;PCR‐confirmed\;(or\;antibody‐positive)\;cohort}{incidence\;rate\;of\;infection\;in\;the\;antibody‐negative\;cohort}$$

### Ethical approvals and study reporting

The research methods were approved by the ethics review boards at HMC (HMC IRB number MRC-05-011) and Weill Cornell Medicine–Qatar (WCM-Q IRB number 20–00017), with waiver of informed consent for the retrospective design of this study. This study is reported as per the Strengthening the Reporting of Observational Studies in Epidemiology (STROBE) guideline (Table A in S1 File). All analyses were originally planned, unless indicated otherwise.

## Results

### Outcome of surveillance system for B.1.1.7

Surveillance for B.1.1.7 cases identified no community transmission cases before 25 December 2020, and very few sporadic cases between 25 December 2020 and 17 January 2021 (Fig 1A). A number of B.1.1.7 cases were identified before 25 December 2020, but only among quarantined incoming travelers. A sudden large jump in community transmission cases was observed on 18 January 2021, and increased transmission continued until the end date of this study, 3 March 2021.

Counts of B.1.1.7 cases demonstrated a classic pattern of exponential epidemic growth for 5 weeks starting from the onset of expansion (Fig 1). The proportion of B.1.1.7 cases among all cases increased rapidly, from negligible before 18 January 2021 to 21% in about 10 days (Fig 1B).

### Viral genome sequencing and analysis

Viral genome sequencing of 46 random S-gene dropout samples confirmed infection with B.1.1.7 in all of them (Fig A in S1 File). These cases exhibited the distinctive characteristics of the Δ69/Δ70 deletion (H69/V70) in the N-terminal domain of the spike protein and the N501Y mutation. In some cases, PCR amplification failed around the N501Y mutation; however, cumulative nucleotide variation at other locations confirmed a high likelihood of a shared haplotype. Sequencing also indicated limited diversity in the viral genome (Fig A in S1 File). Sequencing confirmed the first documented B.1.1.7 community transmission case on 25 December 2020 in a 30-year-old woman with no recent travel history.

### Effective reproduction number (*R*_*t*_)

A sudden, large increase in *R*_*t*_ coincided precisely with the onset of B.1.1.7 expansion (Fig 2A), suggesting higher infectiousness of B.1.1.7, because of either higher transmission probability of infection per contact (for example, because of higher viral load) or longer duration of infectiousness. While *R*_*t*_ averaged 0.97 from 1 August 2020 to 17 January 2021, and was ≥1 only for transient, short durations around specific festivities in Qatar’s multi-national population (Eid al-Adha, Qatar National Day, Christmas, and New Year; Fig 2A) during this period, it was most often well above 1 from 18 January 2021 to 3 March 2021, the end date of this study. Expansion of B.1.1.7 thus triggered the onset of the second wave in Qatar, 7 months after the first wave peaked around 20 May 2020 [1,2]. Comparing the average *R*_*t*_ prior to B.1.1.7 expansion with that for B.1.1.7 after expansion (Fig 2) yielded a ratio of 1.59, suggesting that the B.1.1.7 variant is approximately 60% more infectious than the original variants in Qatar.

### Associations of individual characteristics with infection with B.1.1.7

Characteristics of B.1.1.7 cases and associations with infection are shown in Table 1. The table includes the variables available to study investigators. The odds of infection increased by 3% per day, highlighting the rapid epidemic expansion of this variant.

**Table 1:** Associations of characteristics with infection with B.1.1.7 in individuals tested for SARS-CoV-2 using PCR during the study period (18 January to 3 March 2021).

Characteristic	Tested, *N* (%)	B.1.1.7 variant cases, *N* (%^a^)	Univariable regression analysis		F test, *p*-value	Multivariable regression analysis	
			OR^a^ (95% CI)	*p*-Value		AOR^a^ (95% CI)	*p*-Value
**Sex**							
Men	178,457 (69.6)	5,090 (2.9)	1.00		<0.001	1.00	
Women	78,052 (30.4)	3,393 (4.3)	1.55 (1.48–1.62)	<0.001		1.29 (1.23–1.36)	<0.001
**Age (years)**							
30–39^b^	81,760 (31.9)	2,966 (3.6)	1.00		<0.001	1.00	
<10	28,791 (11.2)	502 (1.7)	0.47 (0.43–0.52)	<0.001		0.35 (0.32–0.39)	<0.001
10–19	26,847 (10.5)	965 (3.6)	0.99 (0.92–1.07)	0.800		0.69 (0.63–0.75)	<0.001
20–29	54,982 (21.4)	1,495 (2.7)	0.74 (0.70–0.79)	<0.001		0.84 (0.79–0.89)	<0.001
40–49	42,205 (16.5)	1,671 (4.0)	1.10 (1.03–1.16)	0.004		1.01 (0.94–1.07)	0.871
50–59	15,970 (6.2)	651 (4.1)	1.13 (1.04–1.23)	0.006		1.03 (0.94–1.12)	0.580
60–69	4,541 (1.8)	188 (4.1)	1.15 (0.99–1.33)	0.074		1.01 (0.86–1.18)	0.897
70–79	1,064 (0.4)	39 (3.7)	1.01 (0.73–1.39)	0.948		0.86 (0.61–1.19)	0.355
80+	349 (0.1)	6 (1.7)	0.46 (0.21–1.04)	0.063		0.32 (0.14–0.71)	0.006
**Nationality**							
Qatari	26,766 (10.4)	916 (3.4)	1.00			1.00	
Pakistani	12,250 (4.8)	490 (4.0)	1.18 (1.05–1.32)	0.005	<0.001	1.93 (1.71–2.17)	<0.001
Filipino	19,398 (7.6)	1,098 (5.7)	1.69 (1.55–1.85)	<0.001		1.69 (1.53–1.86)	<0.001
Indian	72,374 (28.2)	2,403 (3.3)	0.97 (0.90–1.05)	0.428		1.60 (1.46–1.74)	<0.001
Sri Lankan	5,863 (2.3)	278 (4.7)	1.40 (1.22–1.61)	<0.001		1.45 (1.26–1.68)	<0.001
Egyptian	15,423 (6.0)	616 (4.0)	1.17 (1.06–1.30)	0.003		1.38 (1.24–1.54)	<0.001
Sudanese	5,912 (2.3)	167 (2.8)	0.82 (0.69–0.97)	0.020		0.98 (0.82–1.16)	0.805
Nepalese	21,405 (8.3)	390 (1.8)	0.52 (0.46–0.59)	<0.001		0.73 (0.64–0.83)	<0.001
Bangladeshi	18,025 (7.0)	245 (1.4)	0.39 (0.34–0.45)	<0.001		0.43 (0.37–0.50)	<0.001
All other nationalities^c^	59,093 (23.0)	1,880 (3.2)	0.93 (0.86–1.00)	0.066		1.30 (1.19–1.41)	<0.001
**Reason for testing**							
Survey	53,474 (20.8)	1,976 (3.7)	1.00		<0.001	1.00	
Clinical suspicion	28,360 (11.1)	3,160 (11.1)	3.27 (3.08–3.46)	<0.001		2.84 (2.67–3.02)	<0.001
Contact tracing	26,374 (10.3)	1,225 (4.6)	1.27 (1.18–1.37)	<0.001		1.24 (1.15–1.34)	<0.001
Healthcare routine testing	29,195 (11.4)	1,190 (4.1)	1.11 (1.03–1.19)	0.006		1.03 (0.95–1.11)	0.485
Other survey testing	798 (0.3)	30 (3.8)	1.02 (0.70–1.47)	0.924		0.87 (0.60–1.26)	0.469
Individual request	13,507 (5.3)	463 (3.4)	0.93 (0.83–1.03)	0.138		0.76 (0.69–0.85)	<0.001
Pre-travel	27,013 (10.5)	136 (0.5)	0.13 (0.11–0.16)	<0.001		0.12 (0.10–0.14)	<0.001
Port of entry	77,788 (30.3)	303 (0.4)	0.10 (0.09–0.12)	<0.001		0.08 (0.07–0.09)	<0.001
**PCR date**	256,509 (100.0)	8,483 (3.3)	1.031 (1.029–1.033)	<0.001	<0.001	1.031 (1.029–1.032)	<0.001

AOR, adjusted odds ratio; CI, confidence interval; OR, odds ratio.

^a^Estimates are proportion with the B.1.1.7 variant among those tested.

^b^The category with the largest sample size was selected as the reference category.

^c^These include 103 other nationalities of individuals residing in Qatar.

Women had 1.29-fold higher odds of infection than men. Children <10 years of age had 0.35-fold lower odds of being infected than those 30–39 years of age. There were large differences in the odds of infection by nationality, with Bangladeshi and Nepalese individuals having the lowest odds of infection.

### Reinfections in the cohort of individuals with prior PCR-confirmed infections

**Fig 3:**
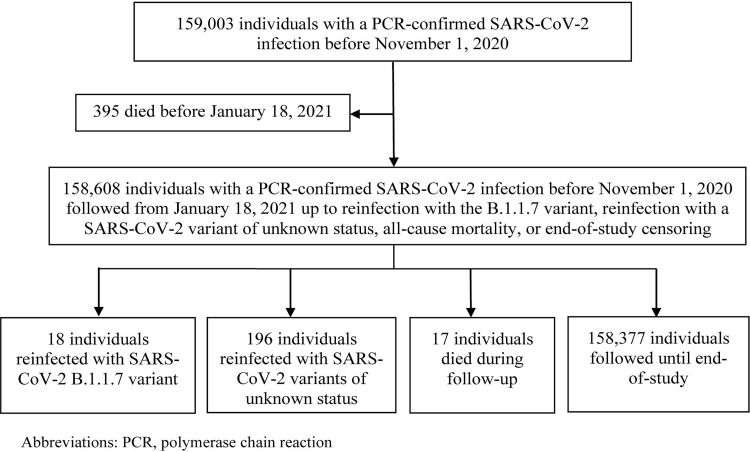
Flow chart of SARS-CoV-2 reinfections with B.1.1.7 and variants of unknown status in the national cohort of individuals with prior PCR-confirmed infections.

Fig 3 shows the process for identifying reinfections with B.1.1.7 and variants of unknown status in the national cohort of individuals with prior PCR-confirmed infections. Of the 158,608 individuals with prior PCR-confirmed infections, 18 were reinfected with B.1.1.7, 196 were reinfected with a variant of unknown status, 17 died during follow-up, and the rest were followed until the end of the study.

The risk of documented reinfection with B.1.1.7 was 0.01% (95% CI: 0.007% to 0.02%) (18 reinfection events in 158,608 individuals with prior PCR-confirmed infections). The incidence rate of documented reinfection was 0.18 (95% CI: 0.11 to 0.29) per 10,000 person-weeks (18 reinfection events in 996,341.5 person-weeks of follow-up).

The risk of documented reinfection with a variant of unknown status was 0.12% (95% CI: 0.11% to 0.14%) (196 reinfection events in 158,608 individuals with prior PCR-confirmed infections). The incidence rate of documented reinfection was 1.97 (95% CI: 1.71 to 2.26) per 10,000 person-weeks (196 reinfection events in 996,341.5 person-weeks of follow-up).

### Reinfections in the cohort of antibody-positive individuals

**Fig 4:**
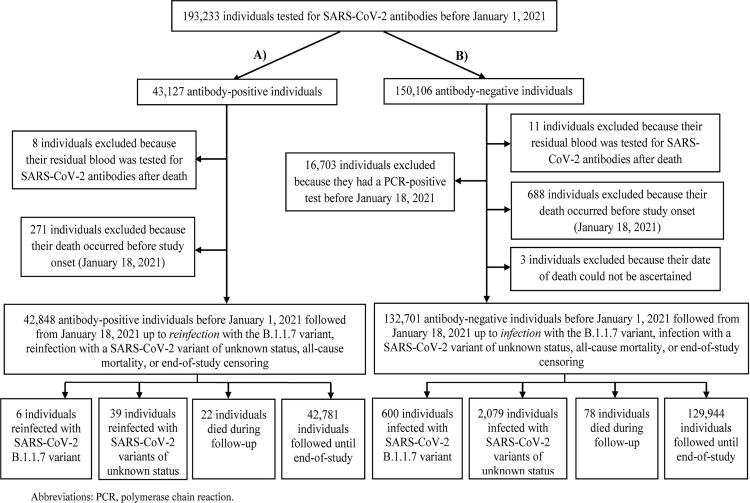
Flow chart of SARS-CoV-2 reinfections with B.1.1.7 and variants of unknown status in the national cohort of antibody-positive individuals and the national cohort of antibody-negative individuals. (A) Antibody-positive individuals. (B) Antibody-negative individuals.

Fig 4 shows the process for identifying reinfections with B.1.1.7 and variants of unknown status in the national cohort of antibody-positive individuals. Of the 42,848 antibody-positive individuals, 6 were reinfected with B.1.1.7, 39 were reinfected with a variant of unknown status, 22 died during follow-up, and the rest were followed until the end of the study.

The risk of documented reinfection with B.1.1.7 was 0.01% (95% CI: 0.005% to 0.03%) (6 reinfection events in 42,848 antibody-positive individuals). The incidence rate of documented reinfection was 0.22 (95% CI: 0.10 to 0.50) per 10,000 person-weeks (6 reinfection events in 269,131.6 person-weeks of follow-up).

The risk of documented reinfection with a variant of unknown status was 0.09% (95% CI: 0.06% to 0.12%) (39 reinfection events in 42,848 antibody-positive individuals). The incidence rate of documented reinfection was 1.45 (95% CI: 1.06 to 1.98) per 10,000 person-weeks (39 reinfection events in 269,131.6 person-weeks of follow-up).

### Comparator cohort: Infections in the cohort of antibody-negative individuals

Fig 4 shows the process for identifying infections with B.1.1.7 and variants of unknown status in the national cohort of antibody-negative individuals. Of the 132,701 antibody-negative individuals with no prior PCR-confirmed infection, 600 were infected with B.1.1.7, 2,079 were infected with another variant, 78 died during follow-up, and the rest were followed until the end of the study.

The risk of documented infection with B.1.1.7 was 0.45% (95% CI: 0.42% to 0.49%) (600 infection events in 132,701 antibody-negative individuals). The incidence rate of documented infection was 7.26 (95% CI: 6.70 to 7.87) per 10,000 person-weeks (600 infection events in 826,222.6 person-weeks of follow-up).

The risk of documented infection with a variant of unknown status was 1.57% (95% CI: 1.50% to 1.63%) (2,079 infection events in 132,701 antibody-negative individuals). The incidence rate of documented infection was 25.16 (95% CI: 24.10 to 26.27) per 10,000 person-weeks (2,079 infection events in 826,222.6 person-weeks of follow-up).

### Comparisons of cohorts and infections

Comparison of the cohort of individuals with prior PCR-confirmed infections with the cohort of antibody-negative individuals showed differences in median age, age distribution, sex, and nationality (Table 2). Comparison of the cohort of antibody-positive individuals with the cohort of antibody-negative individuals also showed differences in age distribution, sex, and nationality (Table 2). These differences largely reflected associations with infection during the first wave in 2020 [1,2,13,14,18].

**Table 2:** Demographic characteristics of study cohorts.

Characteristic	Comparison of prior-PCR-confirmed-infection cohort to antibody-negative cohort			Comparison of antibody-positive cohort to antibody-negative cohort		
	Prior-PCR-confirmed-infection cohort	Antibody-negative cohort	SMD	Antibody-positive cohort	Antibody-negative cohort	SMD
**Number**	158,608	132,701		42,848	132,701	
**Total follow-up time (person-weeks)**	996,341.5	826,222.6		269,131.6	826,222.6	
**Median age (IQR)—years**	34 (27–42)	37 (29–49)	0.34	37 (30–47)	37 (29–49)	0.02
**Age group—*n* (%)**			0.38			0.30
<20 years	17,977 (11.3)	11,983 (9.0)		1,589 (3.71)	11,983 (9.0)	
20–29 years	36,743 (23.2)	23,640 (17.8)		8,600 (20.1)	23,640 (17.8)	
30–39 years	54,506 (34.4)	38,471 (29.0)		14,278 (33.3)	38,471 (29.0)	
40–49 years	30,937 (19.5)	26,545 (20.0)		10,063 (23.5)	26,545 (20.0)	
50–59 years	13,338 (8.4)	17,481 (13.2)		5,635 (13.2)	17,481 (13.2)	
60–69 years	4,068 (2.6)	9,571 (7.2)		2,036 (4.8)	9,571 (7.2)	
70+ years	1,039 (0.7)	5,010 (3.8)		647 (1.5)	5,010 (3.8)	
**Sex**			0.71			0.72
Male	125,291 (79.0)	61,913 (46.7)		33,928 (79.2)	61,913 (46.7)	
Female	33,317 (21.0)	70,788 (53.3)		8,920 (20.8)	70,788 (53.3)	
**Nationality** ^a^			0.79			0.86
Bangladeshi	18,753 (11.8)	5,262 (4.0)		6,778 (15.8)	5,262 (4.0)	
Egyptian	7,621 (4.8)	11,242 (8.5)		2,260 (5.3)	11,242 (8.5)	
Filipino	10,295 (6.5)	9,609 (7.2)		2,152 (5.0)	9,609 (7.2)	
Indian	41,185 (26.0)	21,921 (16.5)		10,185 (23.8)	21,921 (16.5)	
Nepalese	24,387 (15.4)	3,600 (2.7)		6,625 (15.5)	3,600 (2.7)	
Pakistani	9,056 (5.7)	5,107 (3.9)		2,622 (6.1)	5,107 (3.9)	
Qatari	17,846 (11.3)	35,041 (26.4)		4,196 (9.8)	35,041 (26.4)	
Sri Lankan	5,588 (3.5)	2,877 (2.2)		1,467 (3.4)	2,877 (2.2)	
Sudanese	3,675 (2.3)	4,666 (3.5)		1,108 (2.6)	4,666 (3.5)	
Other nationalities^b^	20,202 (12.7)	33,376 (25.2)		5,455 (12.7)	33,376 (25.2)	

IQR, interquartile range; SMD, standardized mean difference.

^a^Nationalities were chosen to represent the most numerous groups in the population of Qatar.

^b^There were 133 other nationalities in the prior-PCR-confirmed-infection cohort, 105 other nationalities in the antibody-positive cohort, and 156 other nationalities in the antibody-negative cohort.

Comparison of reinfections in the prior-PCR-confirmed-infection cohort and the antibody-positive cohort with primary infections in the antibody-negative cohort showed differences in median age, age distribution, sex, nationality, and variant type (Table 3). These differences largely reflected differences in the associations with infection during the second (B.1.1.7) wave, in 2021, versus the first (wild-type) wave, in 2020 [1,2,13,14,18]. The percentage of infections with B.1.1.7 was higher among primary infections than among reinfections.

**Table 3:** Demographic characteristics of individuals with reinfections in the prior-PCR-confirmed-infection and antibody-positive cohorts compared to individuals with primary infections in the antibody-negative cohort.

Characteristic	Comparison of prior-PCR-confirmed-infection cohort to antibody-negative cohort			Comparison of antibody-positive cohort to antibody-negative cohort		
	Reinfections	Primary infections	SMD	Reinfections	Primary infections	SMD
**Number**	214	2,679		45	2,679	
**Median age (IQR)—years**	32 (27–39)	37 (29–46)	0.36	36 (30–44)	37 (29–46)	0.10
**Age group—*n* (%)**			0.42			0.36
<20 years	19 (8.9)	241 (9.0)		1 (2.2)	241 (9.0)	
20–29 years	54 (25.2)	454 (17.0)		9 (20.0)	454 (17.0)	
30–39 years	89 (41.6)	876 (32.7)		17 (37.8)	876 (32.7)	
40–49 years	35 (16.4)	592 (22.1)		8 (17.8)	592 (22.1)	
50–59 years	11 (5.1)	314 (11.7)		6 (13.3)	314 (11.7)	
60–69 years	5 (2.3)	145 (5.4)		2 (4.4)	145 (5.4)	
70+ years	1 (0.5)	57 (2.1)		2 (4.4)	57 (2.1)	
**Sex**			0.81			0.58
Male	175 (81.8)	1,225 (45.7)		33 (73.3)	1,225 (45.7)	
Female	39 (18.2)	1,454 (54.3)		12 (26.7)	1,454 (54.3)	
**Nationality** ^a^			0.55			0.64
Bangladeshi	21 (9.8)	73 (2.7)		6 (13.3)	73 (2.7)	
Egyptian	14 (6.5)	283 (10.6)		2 (4.4)	283 (10.6)	
Filipino	6 (2.8)	148 (5.5)		2 (4.4)	148 (5.5)	
Indian	53 (24.8)	556 (20.8)		12 (26.7)	556 (20.8)	
Nepalese	27 (12.6)	89 (3.3)		4 (8.9)	89 (3.3)	
Pakistani	13 (6.1)	185 (6.9)		2 (4.4)	185 (6.9)	
Qatari	29 (13.6)	565 (21.1)		7 (15.6)	565 (21.1)	
Sri Lankan	3 (1.4)	35 (1.3)		1 (2.2)	35 (1.3)	
Sudanese	8 (3.7)	100 (3.7)		0 (0.0)	100 (3.7)	
Other nationalities^b^	40 (18.7)	645 (24.1)		9 (20.0)	645 (24.1)	
**Variant type**			0.39			0.24
B.1.1.7	18 (8.4)	600 (22.4)		6 (13.3)	600 (22.4)	
Variant of unknown status	196 (91.6)	2,079 (77.6)		39 (86.7)	2,079 (77.6)	

IQR, interquartile range; SMD, standardized mean difference.

^a^Nationalities were chosen to represent the most numerous groups in the population of Qatar.

^b^There were 20 other nationalities among reinfected individuals in the prior-PCR-confirmed-infection cohort, 5 other nationalities among reinfected individuals in the antibody-positive cohort, and 59 other nationalities among infected individuals in the antibody-negative cohort.

### Efficacy of natural infection against reinfection with B.1.1.7 or a variant of unknown status

The efficacy of natural infection against reinfection was estimated by comparing the incidence rate of reinfection in the cohort of individuals with prior PCR-confirmed infections (and in the cohort of antibody-positive individuals) to the incidence rate of infection in the comparator cohort of antibody-negative individuals.

Accordingly, the efficacy of natural infection against reinfection with B.1.1.7 was estimated at 97.5% (95% CI: 95.7% to 98.6%) among those with a prior PCR-confirmed infection, and at 97.0% (95% CI: 92.5% to 98.7%) among those with a prior antibody-positive result.

Similarly, efficacy of natural infection against reinfection with a variant of unknown status was estimated at 92.2% (95% CI: 90.6% to 93.5%) among those with a prior PCR-confirmed infection, and at 94.2% (95% CI: 91.8% to 96.0%) among those with a prior antibody-positive result.

## Discussion

After several months of low SARS-CoV-2 incidence in Qatar, a second wave emerged with the introduction and expansion of the B.1.1.7 variant. This finding suggests a higher transmissibility and/or duration of infectiousness associated with B.1.1.7, as suggested elsewhere [46,47], although some studies have suggested otherwise [48,49]. This finding also supports, in the context of the higher severity of this variant [32,50,51], the global risk associated with variants of concern, which can ignite new epidemic waves once introduced into a naïve population, even with public health restrictions and relatively high levels of natural immunity, as in the case of Qatar [13,14]. Notably, despite the initial rapid spread of the B.1.1.7 variant, this variant was replaced as the dominant variant in subsequent weeks by the B.1.351 (or Beta [23]) variant shortly after it was introduced in mid-February 2021 [7,8,26–28].

Strikingly, evidence of prior infection with an originally circulating (wild-type) variant, regardless of whether this prior infection was ascertained by PCR or antibody testing, was associated with low infection incidence by B.1.1.7 or other circulating variants. The estimated efficacy of natural infection against reinfection was also similar to that estimated earlier for these 2 cohorts, before introduction of any variants of concern, at about 95% [16,17], and similar to that estimated in other global studies [52]. These findings are consistent with studies indicating that B.1.1.7 is not associated with serious immune evasion [53–55]. They also suggest that there is no appreciable waning of immunity even among those who acquired the infection in the first wave nearly a year ago.

There were large differences in the risk of infection with B.1.1.7 by sex and nationality. These differences, however, could be explained by differential exposure to SARS-CoV-2 in the first wave versus the second (B.1.1.7) wave. The first wave primarily affected craft and manual workers, who are typically single, young men of Bangladeshi and Nepalese nationality [1–3,13,14,18]. At end of this wave, seroprevalence was estimated at about 60% among these workers [13,14]. Women had a low exposure risk during the first wave, and their seroprevalence was estimated at <10% [18]. Therefore, subpopulations that escaped exposure during the first wave were most affected in the second wave, while subpopulations most affected in the first wave were protected against reinfection with B.1.1.7. In both waves, however, children <10 years of age were less likely to be infected than older individuals, with B.1.1.7 or any other variant [1,18], possibly because of the nature of social networks in Qatar, or because of lower biological susceptibility and rapid infection clearance [56–58].

This study has limitations. To ensure no false-positive cases, B.1.1.7 cases were defined as having a PCR Ct value ≤ 25 for both the N and ORF1ab genes but a negative outcome for the S gene. While this definition prevented false-positive cases, and indeed all sequenced cases were confirmed B.1.1.7 cases, it likely underestimated the actual number of B.1.1.7 cases, that is, whenever a case had a PCR Ct value > 25 for either the N or the ORF1ab gene, but a negative outcome for the S gene. This limitation, however, is not likely to appreciably affect derived relative epidemiological measures, such as efficacy of natural infection against reinfection and *R*_*t*_, as this limitation should affect numerators and denominators proportionally.

The efficacy of natural infection against reinfection was assessed against documented PCR-confirmed reinfections, but other reinfections could have occurred and gone undocumented, perhaps because of minimal/mild or no symptoms. Thus, the estimated efficacy may not similarly hold against milder forms of reinfection. During the course of follow-up, some antibody-negative persons may have developed antibodies due to infections that were not documented by PCR, and these individuals could be misclassified in the comparator cohort. Antigen testing was limited in Qatar during the study duration, and positive results had to be confirmed by PCR testing for individuals to be considered cases; therefore, it is not likely that reinfections diagnosed through antigen testing were missed.

Travel history was not available to investigate whether there was any loss to follow-up due to travel out of the country. Imperfect PCR or antibody assay sensitivity and specificity may have affected current or prior infection ascertainment. However, all PCR and serological testing was performed using extensively used, investigated, and validated quality commercial platforms with essentially 100% sensitivity and specificity (Text A in S1 File). The investigated observational cohorts were not blinded, randomized, or matched, and thus there remains potential for residual uncontrolled confounding.

In conclusion, the introduction of B.1.1.7 into a population can result in a new epidemic wave, as documented here for Qatar. This highlights the global risks posed by circulation of variants of concern and how these can undermine the global response to this pandemic. However, natural immunity in those previously infected was strongly associated with limited reinfection incidence by B.1.1.7 or other variants.

## Supporting information

S1 File Text A: Laboratory methods. Text B: Viral genome sequencing and analysis. Table A: STROBE checklist for cohort studies. Fig A: Viral genome sequencing analysis of 46 random S-gene dropout samples.
(DOCX)

## Abbreviations

Ct: cycle threshold
HMC: Hamad Medical Corporation
OR: odds ratio
RT-qPCR: quantitative reverse transcription PCR
SMD: standardized mean difference
