# Supplementary material for: Introduction and expansion of the SARS-CoV-2 B.1.1.7 variant and reinfections in Qatar: A nationally representative cohort study
Source: PLoS Med. 2021 Dec 16;18(12):e1003879. doi: 10.1371/journal.pmed.1003879 (PMC8726501; doi:10.1371/journal.pmed.1003879)
Supplement: S1 File — Text A: Laboratory methods. Text B: Viral genome sequencing and analysis. Table A: STROBE checklist for cohort studies. Fig A: Viral genome sequencing analysis of 46 random S-gene dropout samples. (DOCX) [file pmed.1003879.s001.docx]

Supporting information

Table of Contents

[Text A. Laboratory methods 2](#_Toc89348483)

[Text B. Viral genome sequencing and analysis 3](#_Toc89348484)

[Table A. STROBE checklist for cohort studies. 5](#_Toc89348485)

[Fig A. Viral genome sequencing analysis of 46 random S-gene dropout samples. While some genomes did not sequence well at the N501Y location (23063), a cumulation of other variants strongly argue that the haplotype is shared with other N501Y containing genomes. 7](#_Toc89348486)

[References 8](#_Toc89348487)

# Text A. Laboratory methods

Nasopharyngeal and/or oropharyngeal swabs were collected for PCR testing and placed in Universal Transport Medium (UTM). Aliquots of UTM were: extracted on a QIAsymphony platform (QIAGEN, USA) and tested with real-time reverse-transcription PCR (RT-qPCR) using TaqPath COVID-19 Combo Kits (100% sensitivity and specificity [1]; Thermo Fisher Scientific, USA) on an ABI 7500 FAST (ThermoFisher, USA); extracted using a custom protocol [2] on a Hamilton Microlab STAR (Hamilton, USA) and tested using AccuPower SARS-CoV-2 Real-Time RT-PCR Kits (100% sensitivity and specificity [3]; Bioneer, Korea) on an ABI 7500 FAST; or loaded directly into a Roche cobas 6800 system and assayed with a cobas SARS-CoV-2 Test (95% sensitivity, 100% specificity [4]; Roche, Switzerland). The first assay targets the viral S, N, and ORF1ab regions. The second targets the viral RdRp and E-gene regions, and the third targets the ORF1ab and E-gene regions.

Antibodies against SARS‑CoV‑2 in serological samples were detected using a Roche Elecsys Anti‑SARS‑CoV‑2 assay (99.5% sensitivity [5], 99.8% specificity [5, 6]; Roche, Switzerland), an electrochemiluminescence immunoassay that uses a recombinant protein representing the nucleocapsid (N) antigen for antibody binding. Results were interpreted according to the manufacturer’s instructions (reactive: optical density (proxy for antibody titer [7]) cutoff index ≥1.0 vs. non-reactive: optical density cutoff index <1.0).

All tests were analyzed at the HMC Central Laboratory or at Sidra Medicine Laboratory, following standardized protocols.

# Text B. Viral genome sequencing and analysis

Viral genome sequencing was conducted on a subset of S-gene “dropout” samples to confirm B.1.1.7. Viral RNA was extracted using Quick-RNA Viral Kits (Zymo Research, Irvine, USA; Cat. No. R1041) and eluted in 30 µL of nuclease-free water. RNA quality was assessed with real-time quantitative polymerase chain reaction (RT-qPCR) using SARS-CoV-2 (2019-nCoV) CDC qPCR Probe Assay Research Use Only (RUO) kits (Integrated DNA Technologies, USA; Cat number 10006713) and Luna Universal Probe One-Step RT-qPCR Kits (New England BioLabs, USA; Cat number E3006E) on an Applied Biosystems 7500 Fast Real-Time PCR instrument (Applied Biosystems, CA, USA).

Next-generation sequencing (NGS) library construction was performed using CleanPlex SARS-CoV-2 Panels (Paragon Genomics, USA; SKU: 918012). Gel-size selection on 3% agarose gels was utilized to prevent formation of adapter dimers. NGS libraries were quantified using KAPA Library Quantification Kits (Roche, USA; KK4824), and normalized, pooled, and sequenced on an Illumina MiSeq instrument using paired-end 150-bp kits (Illumina, USA; MS-102-2002). All procedures were implemented following manufacturers’ protocols.

Raw sequences were processed with CUTADAPT (v2·10) [8] to exclude contaminating adapter sequences. Adapter trimming was performed using parameters -g CCTACACGACGCTCTTCCGATCT **-a** AGATCGGAAGAGCACACGTCTGAA **-A** AGATCGGAAGAGCGTCGTGTAGG **-G** TTCAGACGTGTGCTCTTCCGATCT **-e** 0·1 **-O** 9 **-m** 50 **-n** 2. Only paired reads with minimum length of 50 bp were retained for analysis. The latter filtered reads were aligned to the SARS-CoV-2 reference genome (NC_045512) using BWA-MEM.FGBIO (v1·3·0) [9] was subsequently used to remove PCR primer sequences from the resulting BAM file.

Variant calling and genotyping were performed with VarScan multi-sample mpileup [10] with the pileup file generated using SAMTOOLS mpileup (v1·10) [11] with --min-BQ 20 and --min-MQ 20 parameters. The mpileup2snp function of VarScan was then applied with the filtering parameters --min-var-freq 0·2, --min-coverage 5, and --min-avg-qual 20, to generate the final VCF file.

# Table A. STROBE checklist for cohort studies.

|  | Item No | Recommendation | Main Text |
| --- | --- | --- | --- |
| **Title and abstract** | 1 | (*a*) Indicate the study’s design with a commonly used term in the title or the abstract | Title & Abstrat |
|  |  | (*b*) Provide in the abstract an informative and balanced summary of what was done and what was found | Abstract |
| Introduction | | | |
| Background/rationale | 2 | Explain the scientific background and rationale for the investigation being reported | Author summary & Introduction, paragraphs 1-3 |
| Objectives | 3 | State specific objectives, including any prespecified hypotheses | Introduction, paragraph 3 |
| Methods | | | |
| Study design | 4 | Present key elements of study design early in the paper | Methods (‘Sources of data’ & ‘Surveillance system for B.1.1.7’) |
| Setting | 5 | Describe the setting, locations, and relevant dates, including periods of recruitment, exposure, follow-up, and data collection | Methods (‘Sources of data’ to ‘Assessment of reinfection with B.1.1.7 and variants of unknown status’) |
| Participants | 6 | (*a*) Give the eligibility criteria, and the sources and methods of selection of participants. Describe methods of follow-up | Methods (‘Sources of data’ to ‘Assessment of reinfection with B.1.1.7 and variants of unknown status’) & Figs. 3-4 |
|  |  | (*b*) For matched studies, give matching criteria and number of exposed and unexposed | NA |
| Variables | 7 | Clearly define all outcomes, exposures, predictors, potential confounders, and effect modifiers. Give diagnostic criteria, if applicable | Methods, S1 Text, & S2 Text |
| Data sources/ measurement | 8* | For each variable of interest, give sources of data and details of methods of assessment (measurement). Describe comparability of assessment methods if there is more than one group | Methods (‘Sources of data’ to ‘Assessment of reinfection with B.1.1.7 and variants of unknown status’), S1 Text, & S2 Text |
| Bias | 9 | Describe any efforts to address potential sources of bias | Methods (‘Effective reproduction number and infectiousness of B.1.1.7’ to ‘Assessment of reinfection with B.1.1.7 and variants of unknown status’) |
| Study size | 10 | Explain how the study size was arrived at | Figs. 3-4 |
| Quantitative variables | 11 | Explain how quantitative variables were handled in the analyses. If applicable, describe which groupings were chosen and why | Tables 1-3 |
| Statistical methods | 12 | (*a*) Describe all statistical methods, including those used to control for confounding | Methods (‘Effective reproduction number and infectiousness of B.1.1.7’ to ‘Comparator antibody-negative group and efficacy of natural infection against reinfection’) |
|  |  | (*b*) Describe any methods used to examine subgroups and interactions | NA |
|  |  | (*c*) Explain how missing data were addressed | NA, see Methods (‘Sources of data’) |
|  |  | (*d*) If applicable, explain how loss to follow-up was addressed | NA |
|  |  | (*e*) Describe any sensitivity analyses | NA |
| Results | | |  |
| Participants | 13* | (a) Report numbers of individuals at each stage of study—eg numbers potentially eligible, examined for eligibility, confirmed eligible, included in the study, completing follow-up, and analysed | Figs. 3-4 |
|  |  | (b) Give reasons for non-participation at each stage |  |
|  |  | (c) Consider use of a flow diagram |  |
| Descriptive data | 14 | (a) Give characteristics of study participants (eg demographic, clinical, social) and information on exposures and potential confounders | Results (‘Associations with infection with B.1.1.7’) & Table 2 |
|  |  | (b) Indicate number of participants with missing data for each variable of interest | NA, see Methods (‘Sources of data’) |
|  |  | (c) Summarise follow-up time (eg, average and total amount) | Results (‘Reinfections in the cohort of individuals with prior PCR-confirmed infections’ to ‘Comparator cohort: Infections in the cohort of antibody-negative individuals’) & Table 2 |
| Outcome data | 15 | Report numbers of outcome events or summary measures over time | Results (‘Reinfections in the cohort of individuals with prior PCR-confirmed infections’ to ‘Efficacy of natural infection against reinfection with B.1.1.7 or variant of unknown status’), Figs. 3-4 & Tables 1 & 3 |
| Main results | 16 | (a) Give unadjusted estimates and, if applicable, confounder-adjusted estimates and their precision (eg, 95% confidence interval). Make clear which confounders were adjusted for and why they were included | Results (‘Reinfections in the cohort of individuals with prior PCR-confirmed infections’ to ‘Efficacy of natural infection against reinfection with B.1.1.7 or variant of unknown status’) & Table 1 |
|  |  | (b) Report category boundaries when continuous variables were categorized | Tables 1-3 |
|  |  | (c) If relevant, consider translating estimates of relative risk into absolute risk for a meaningful time period | NA |
| Other analyses | 17 | Report other analyses done—eg analyses of subgroups and interactions, and sensitivity analyses | NA |
| Discussion | | | |
| Key results | 18 | Summarise key results with reference to study objectives | Discussion, paragraphs 1-3 |
| Limitations | 19 | Discuss limitations of the study, taking into account sources of potential bias or imprecision. Discuss both direction and magnitude of any potential bias | Discussion, paragraphs 4-6 |
| Interpretation | 20 | Give a cautious overall interpretation of results considering objectives, limitations, multiplicity of analyses, results from similar studies, and other relevant evidence | Discussion, paragraph 7 |
| Generalisability | 21 | Discuss the generalisability (external validity) of the study results | Discussion, paragraphs 4-6 |
| Other information | | | |
| Funding | 22 | Give the source of funding and the role of the funders for the present study and, if applicable, for the original study on which the present article is based | Funding statement |

Abbreviations: NA: not applicable.

Fig A. Viral genome sequencing analysis of 46 random S-gene dropout samples. While some genomes did not sequence well at the N501Y location (23063), a cumulation of other variants strongly argue that the haplotype is shared with other N501Y containing genomes.


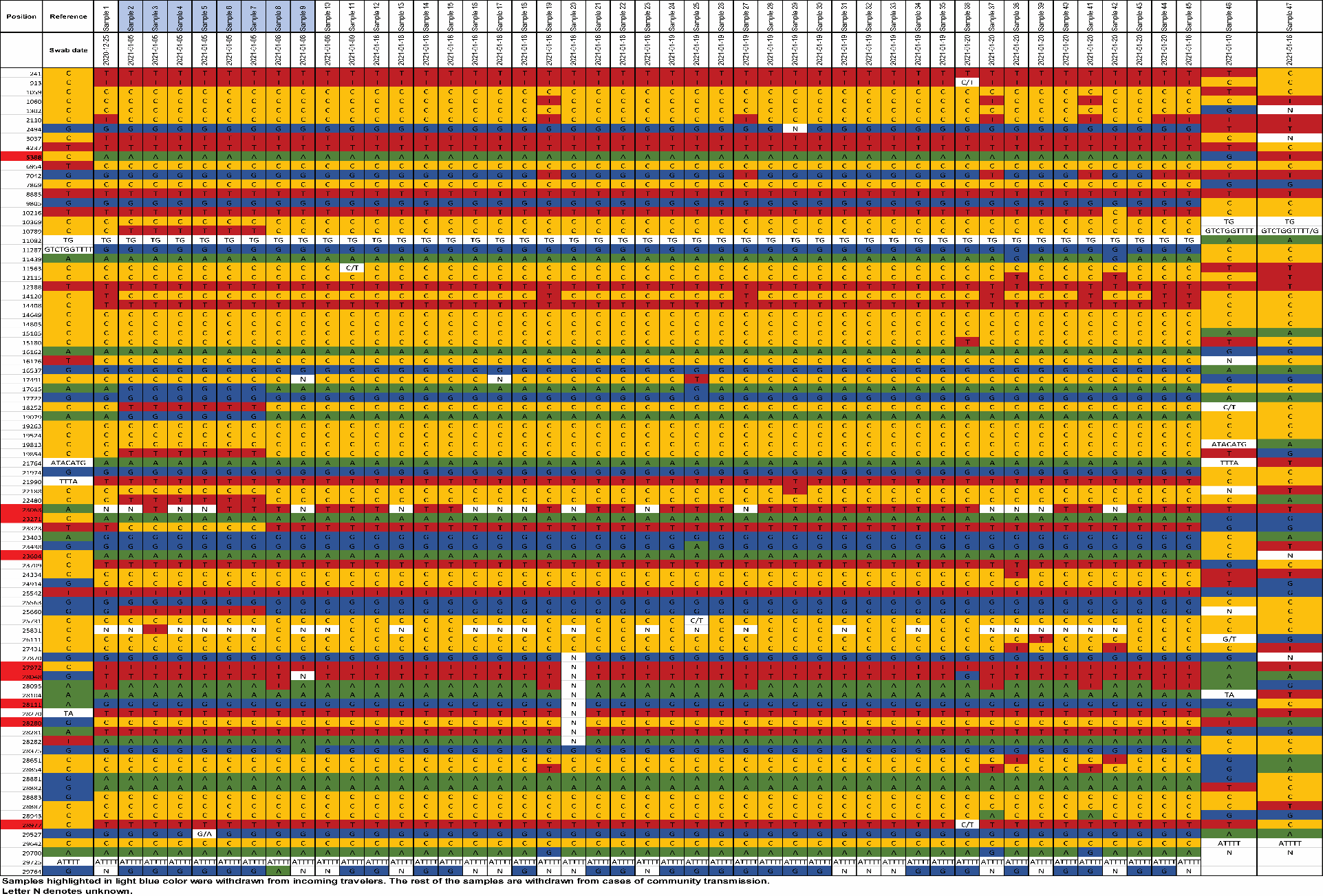


References

1. Thermo Fisher Scientific. TaqPath COVID‑19 CE‑IVD RT‑PCR Kit instructions for use. 2020 [cited 2020 Dec 2]. Available from: <https://assets.thermofisher.com/TFS-Assets/LSG/manuals/MAN0019215_TaqPathCOVID-19_CE-IVD_RT-PCR%20Kit_IFU.pdf>.

2. Kalikiri MKR, Hasan MR, Mirza F, Xaba T, Tang P, Lorenz S. High-throughput extraction of SARS-CoV-2 RNA from nasopharyngeal swabs using solid-phase reverse immobilization beads. medRxiv.2020.04.08.20055731 [preprint]. 2020 [cited 2021 Sep 20]. Available from: <https://www.medrxiv.org/content/10.1101/2020.04.08.v1>.

3. Kubina R, Dziedzic A. Molecular and Serological Tests for COVID-19 a Comparative Review of SARS-CoV-2 Coronavirus Laboratory and Point-of-Care Diagnostics. Diagnostics (Basel). 2020;10(6). doi: 10.3390/diagnostics10060434.

4. US Food and Drug Administration. Cobas SARS-CoV-2: Qualitative assay for use on the cobas 6800/8800 Systems. 2020 [cited 2020 Dec 02]. Available from: <https://www.fda.gov/media/136049/download>

5. Muench P, Jochum S, Wenderoth V, Ofenloch-Haehnle B, Hombach M, Strobl M, et al. Development and Validation of the Elecsys Anti-SARS-CoV-2 Immunoassay as a Highly Specific Tool for Determining Past Exposure to SARS-CoV-2. J Clin Microbiol. 2020;58(10). doi: 10.1128/JCM.01694-20.

6. The Roche Group. Roche’s COVID-19 antibody test receives FDA Emergency Use Authorization and is available in markets accepting the CE mark. 2020 [cited 2021 Jun 5]. Available from: <https://www.roche.com/media/releases/med-cor-2020-05-03.htm>.

7. Oved K, Olmer L, Shemer-Avni Y, Wolf T, Supino-Rosin L, Prajgrod G, et al. Multi-center nationwide comparison of seven serology assays reveals a SARS-CoV-2 non-responding seronegative subpopulation. EClinicalMedicine. 2020;29:100651. doi: 10.1016/j.eclinm.2020.100651.

8. Martin M. Cutadapt removes adapter sequences from high-throughput sequencing reads. ISSN 2226-6089. 2011 [cited 2020 Sep 17]. Available from: <http://journal.embnet.org/index.php/embnetjournal/article/view/200>. EMBnetjournal. 2011;17(1):10-2. doi:<https://doi.org/.14806/ej.17.1.200>.

9. Li H, Durbin R. Fast and accurate short read alignment with Burrows-Wheeler transform. Bioinformatics. 2009;25(14):1754-60. doi: 10.1093/bioinformatics/btp324.

10. Koboldt DC, Chen K, Wylie T, Larson DE, McLellan MD, Mardis ER, et al. VarScan: variant detection in massively parallel sequencing of individual and pooled samples. Bioinformatics. 2009;25(17):2283-5. doi: 10.1093/bioinformatics/btp373.

11. Li H, Handsaker B, Wysoker A, Fennell T, Ruan J, Homer N, et al. The Sequence Alignment/Map format and SAMtools. Bioinformatics. 2009;25(16):2078-9. doi: 10.1093/bioinformatics/btp352.
